# Weighted Feature Gaussian Kernel SVM for Emotion Recognition

**DOI:** 10.1155/2016/7696035

**Published:** 2016-10-11

**Authors:** Wei Wei, Qingxuan Jia

**Affiliations:** School of Automation, Beijing University of Posts and Telecommunications, Beijing 100876, China

## Abstract

Emotion recognition with weighted feature based on facial expression is a challenging research topic and has attracted great attention in the past few years. This paper presents a novel method, utilizing subregion recognition rate to weight kernel function. First, we divide the facial expression image into some uniform subregions and calculate corresponding recognition rate and weight. Then, we get a weighted feature Gaussian kernel function and construct a classifier based on Support Vector Machine (SVM). At last, the experimental results suggest that the approach based on weighted feature Gaussian kernel function has good performance on the correct rate in emotion recognition. The experiments on the extended Cohn-Kanade (CK+) dataset show that our method has achieved encouraging recognition results compared to the state-of-the-art methods.

## 1. Introduction

Emotion recognition has necessary applications in the real world. Its applications include but are not limited to artificial intelligence and human computer interaction. It remains a challenging and attractive topic. There are many methods which have been proposed for handling problems in emotion recognition. Speech [1, 2], physiological [3–5], and visual signals have been explored for emotion recognition. Speech signals are discontinuous signals, since they can be captured only when people are talking. Acquirement of physiological signal needs some special physiological sensors. Visual signal is the best choice for emotion recognition based on the above reasons. Although the visual information provided is useful, there are challenges regarding how to utilize this information reliably and robustly. According to Albert Mehrabian's 7%–38%–55% rule, facial expression is an important mean of detecting emotions [6].

Further studies have been carried out on emotion recognition problems in facial expression images during the last decade [7, 8]. Given a facial expression image, estimate the correct emotional state, such as anger, happiness, sadness, and surprise. The general process has two steps: feature extraction and classification. For feature extraction, geometric feature, texture feature, motion feature, and statistical feature are in common use. For classification, methods based on machine learning algorithm are frequently used. According to speciality of features, applying weighted features to machine learning algorithm has become an active research topic.

In recent years, emotion recognition with weighted feature based on facial expression has become a new research topic and received more and more attention [9, 10]. The aim is to estimate emotion type from a facial expression image captured during physical facial expression process of a subject. But the emotion features captured from the facial expression image are strongly linked to not the whole face but some specific regions in the face. For instance, features of eyebrow, eye, nose, and mouth areas are closely related to facial expression [11]. Besides, the effect of each feature on recognition result is different. In order to make the best of feature, using feature weighting technique can further enhance recognition performance. While there are several approaches of confirming weight, it remains an open issue on how to select feature and calculate corresponding weight effectively.

In this paper, a new emotion recognition method based on weighted feature facial expression is presented. It is motivated by the fact that emotion can be described by facial expression and each facial expression feature has different impact on recognition results. Different from previous works by calculating weight of each feature directly, this method considers impact of feature by calculating subrecognition rate. Our method consists of two stages: weight calculation stage and recognition stage. In the weight calculation stage, we first divide face into 4 areas according to degree of facial behavior changes. Then, we use each area's features to calculate corresponding recognition rate. At last, we calculate weight of each area's features according to magnitude of recognition rate. In the recognition stage, we first use the above weight results to calculate weighted kernel function. Then, we obtain a new recognition model based on SVM with weighted kernel function.

For the proposed method, there are three main contributions and differences compared to the preliminary work. (1) A more advanced weight of feature method is used. In previous method, the weight of each feature was calculated individually without practical verification. To overcome this shortage, we group features and calculate corresponding subrecognition rate. Then we calculate weight of feature groups based on their respective subrecognition rate. (2) In the recognition stage, the previous method used the weight of features directly. In this paper, we use weight of feature groups to weight kernel function. Then we use new weighted kernel function in machine learning model. (3) The proposed method has been evaluated in a database which contains 7 kinds of emotions. Moreover, comparison results have been carefully analyzed and studied on whether to use weighted kernel function. The rest of the paper is organized as follows: Section 2 gives an overview of related works on feature extraction of facial expression, calculation of weight of feature, and classification of emotion.  Section 3 describes the theorem in proposed method and proofs.  Section 4 verifies the proposed method by experiment and analyzes experimental results.  Section 5 concludes the paper.

## 2. Related Work

The recognition performance of motion based methods is highly dependent on the feature extraction methods. Many novel approaches have been proposed for feature extraction based on facial expression. They can be broadly classified into two categories: appearance-based methods and geometric-based methods. The appearance-based methods extract intensity or other texture features from facial expression images. The common methods of feature extraction include Local Binary Patterns (LBP) [12, 13], Histogram of Oriented Gradient (HOG) [14, 15], Gabor Wavelet [16, 17], and Scale-Invariant Feature Transform (SIFT) [18, 19]. These features can be used to extract Action Unit (AU) feature and recognize facial expression. The geometric-based methods describe facial component shapes based on key points of facial detected on images, such as eyebrows, eyes, nose, mouth, and contour line. The movement of these key points can be used for guiding the facial expression recognition process. For instance, the active appearance model (AAM) [20] or Active Shape Model (ASM) [21, 22] and the constrained local model (CLM) [23] are widely used to detect and trace these key points of face to record their displacement. However, the location accuracy of both ASM and AAM relies on their geometric face models. And the model training phases sometimes need manual works and are usually time-consuming.

The recognition results obtained by classification algorithm are affected by all features. So the introduction of weight can distinguish the contribution of different features and improve classification performance. A variety of methods have been proposed to calculate the weight of every feature. Reference [24] presented Euclidean metric in the criterion extended to Minkowski metric to calculate weight of each feature directly. Some methods divided the facial image into some uniform subregions and calculated the weight of each subregion. Reference [25] introduced information entropy to distinguish the contribution of different partitions of the face. Reference [26] estimated the weight of each subregion by employing the local variance. For feature weighting in different ways, feature selection and weight calculation might be recognized as a latent problem. One effective method to solve this problem is to perform feature weighting based on the obtained feedback. Some methods [27, 28] divided the facial image into some uniform subregions and returned the subregion result for feature weighting. There is no restriction on each feature, which provides freedom on how the feature representations are structured.

Many machine learning methods have been proposed to classify facial expressions, such as SVM [29], Random Forest (RF) [30], Neural Network (NN) [31], and *K* nearest neighbor (*K*NN) [32]. Reference [33] presented the performance of RF and SVM in classification of facial recognition. Reference [34] used boosting technique for the construction of NNEs and the final prediction is made by Naive Bayes (NB) classifier. Reference [35] divided the region into different types and combined the characteristic of the Fuzzy Support Vector Machine (FSVM) with *K*NN, switching the classification methods to the different types. The studies show that these methods are extremely suitable for facial expression classification.

## 3. Support Vector Machine

### 3.1. Linear Support Vector Machines

SVM is a new supervised learning model with associated learning algorithm for classification problem of data whose ultimate aim is to find the optimal separating hyperplane. The mathematical model of SVM is shown below.

Given a training set ${\left\{y_{i},{\vec{x}}_{i}\right\}}_{i=1}^{l}$, where ${\vec{x}}_{i}\in R^{n}$ is input and *y*_*i*_ ∈ {−1, +1} is the corresponding output, if there is a hyperplane which can divide all the points ${\vec{x}}_{i}$ into two groups correctly, we aim to find the “maximum-margin hyperplane” where the distance between the hyperplane and the nearest point ${\vec{x}}_{i}$ from either group is maximized. By introducing the penalty parameter *c* > 0 and the slack variable $\vec{\xi }=\left(\xi_{1},\xi_{2},\ldots ,\xi_{l}\right)$, the optimal hyperplane can be obtained by solving constraint optimization problem as follows:

(1) min 12ω→2+C∑i=1lξis.t. yiω→·x→i+b≥1,i=1,2,…,l ξi≥0,i=1,2,…,l.

Based on Lagrangian multiplier method, the problem is converted into a dual problem as follows:

(2) max ∑i=1lai−12∑i,j=1laiajyiyjx→i·x→js.t. ∑i=1laiyi=0, 0≤ai≤C,i=1,2,…,l,

where *a*_*i*_ > 0 are the Lagrange multipliers of samples ${\vec{x}}_{i}$. Only a few *a*_*i*_ > 0 are solutions of the problem of removing the parts of *a*_*i*_ = 0, so that we can get the classification decision function as follows:

(3) $$\begin{array}{c} f\left(\;x\;\right)=\mathrm{sign}\left[\;\overset{l}{\underset{i=1}{\sum }}a_{i}y_{i}\left(\;{\vec{x}}_{i}\cdot \vec{x}\;\right)+b\;\right]. \end{array}$$

### 3.2. Nonlinear Support Vector Machines

For the linearly nonseparable problem, we first map the data to some other high-dimensional space *H*, using a nonlinear mapping which we call Φ. Then we use linear model to achieve classification in new space *H*. Through defined “kernel function” *k*, (2) is converted as follows:

(4) max ∑i=1lai−12∑i,j=1laiajyiyjkx→i·x→js.t. ∑i=1laiyi=0, 0≤ai≤C,i=1,2,…,l.

And the corresponding classification decision function is converted as follows:

(5) $$\begin{array}{c} f\left(\;x\;\right)=\mathrm{sign}\left[\;\overset{l}{\underset{i=1}{\sum }}a_{i}y_{i}k\left(\;{\vec{x}}_{i}\cdot \vec{x}\;\right)+b\;\right]. \end{array}$$

The selection of kernel function aims to take the place of inner product of basis function. The ordinary kernel functions investigated for linearly nonseparable problems are as follows:

(1) *n*th-degree polynomial kernel function
  (6) $$\begin{array}{c} k\left(\;{\vec{x}}_{i},{\vec{x}}_{j}\;\right)={\left(\;{\vec{x}}_{i}\cdot {\vec{x}}_{j}+1\;\right)}^{d},\;d=1,2,\ldots \end{array}$$
(2) (Gaussian) radial basis kernel function
  (7) $$\begin{array}{c} k\left(\;{\vec{x}}_{i},{\vec{x}}_{j}\;\right)=\exp\left(\;-\gamma {\left\|\;{\vec{x}}_{i}-{\vec{x}}_{j}\;\right\|}^{2}\;\right) \end{array}$$
(3) Sigmoid kernel function
  (8) $$\begin{array}{c} k\left(\;{\vec{x}}_{i},{\vec{x}}_{j}\;\right)=\tanh\left[\;b\left(\;{\vec{x}}_{i}\cdot {\vec{x}}_{j}\;\right)+c\;\right],\;b>0,\;\;c<0. \end{array}$$

### 3.3. Weighted Feature SVM

Weighted feature SVM is based on weighted kernel function of SVM, which is defined as Definition 1.

**Definition 1 .**

Let *k* be a kernel function defined in *X∗X*, *X*⊆*R* ^*n*^. *P* is a linear transformation square matrix of order *n* of given input space, where *n* is dimensionality of input space. Weighted feature kernel function *k*_*P*_ is defined as

(9) $$\begin{array}{c} k_{P}\left(\;{\vec{x}}_{i},{\vec{x}}_{j}\;\right)=k\left(\;{\vec{x}}_{i}^{T}P,{\vec{x}}_{j}^{T}P\;\right), \end{array}$$

where *P* is referred to as a weighted feature matrix. The different choices for *P* lead to different weight situation:

(1) *P* is an identity matrix of order *n*, which is no weight situation.
(2) *P* is a diagonal matrix of order *n*, where (*P*)_*ii*_ = *ω*_*i*_  (1 ≤ *i* ≤ *n*) is the weight of *i*th feature and not all *ω*_*i*_ are equal to the others
  (10) $$\begin{array}{c} P=\left[\begin{array}{cccc} \omega_{1} & & & \\ & \omega_{2} & & \\ & & \ddots & \\ & & & \omega_{n} \end{array}\right]. \end{array}$$
(3) *P* is an arbitrary matrix of order *n*, which is full weight situation
  (11) $$\begin{array}{c} P=\left[\begin{array}{cccc} \omega_{11} & \omega_{12} & \cdots & \omega_{1n}\\ \omega_{21} & \omega_{22} & \cdots & \omega_{2n}\\ \vdots & \vdots & \ddots & \vdots \\ \omega_{n1} & \omega_{n2} & \cdots & \omega_{nn} \end{array}\right]. \end{array}$$

We only consider *P* is a diagonal matrix of order *n* in this paper.

**Definition 2 .**

The ordinary weighted feature kernel function can be got by (9), and the process is shown as follows:

(1) Weighted feature polynomial kernel function
  (12) kPx→i,x→j=x→iTP·x→jTP+1d=x→iTPPTx→jT+1d,d=1,2,…
(2) Weighted feature (Gaussian) radial basis kernel function
  (13) kPx→i,x→jexp⁡−γx→iTP−x→jTP2=exp⁡−γx→i−x→jTPPTx→i−x→j
(3) Weighted feature sigmoid kernel function
  (14) kPx→i,x→jtanh⁡bx→iTP·x→jTP+c=tanh⁡bx→iTPPTx→jT+c,b>0,  c<0.

The motivation for introducing kernel function is to search nonlinear model in the new feature space which is obtained by using nonlinear mapping. Matrix *P* appears not to be related to the motivation, since it acts as linear mapping. However, it can be useful in practice, because it can change geometry shape of input space and feature space, thereby changing the weight of different functions in the feature space. And the weighted feature Gaussian basis kernel function is still a nonlinear model after using linear transformation. The conclusion can be proved by Theorem 3.

**Theorem 3 .**

Let *k* be a kernel function defined in *X∗X*, *X*⊆*R* ^*n*^. *ϕ* : *X* → *F* is a mapping from input space to feature space. *P* is a linear transformation square matrix and ${\tilde{x}}_{i}={\tilde{x}}_{i}^{T}P$. Then it deduces $\left\|\phi ({\tilde{x}}_{i}-{\tilde{x}}_{j})\right\|\neq \left\|\phi (x_{i}-x_{j})\right\|$.

**Proof**

$k\left(\vec{x},\vec{x}\right)=1$, $\forall \vec{x}$ acts as any of the three ordinary kernel functions in Definition 1; then it deduces

(15) ϕx~i−ϕx~j=ϕx~i−ϕx~j·ϕx~i−ϕx~j=ϕx~i·ϕx~i−2ϕx~i·ϕx~j+ϕx~jϕx~j=kx~i,x~i−2kx~i,x~j+kx~j,x~j=2−2kx~i,x~j=2−2kxiTP,xjTP≠2−2kxi,xj=ϕxi−ϕxj.

**Theorem 4 .**

When there is *ω*_*k*_ = 0  (1 ≤ *k* ≤ *n*), *k*th feature of sample data is irrelevant to calculation of kernel functions and output of classifier. Furthermore, the smaller the value of *ω*_*k*_  (1 ≤ *k* ≤ *n*), the less the effect of calculation of kernel functions and output of classifier.

**Proof**

From definition of weighted feature kernel function and classification decision function (5), the conclusion is straightforward.

Theorem 3 indicates that changes of location relation between spot and spot lead to changes of geometry shape of feature space after linear transformation. And there may be better linear separating hyperplane in new feature space to improve the classification performance of SVM.  Theorem 4 indicates that weighted kernel function can reduce the effects of weak correlation and no-correlative features, and we are looking forward to better classification results. The experiment results in the following section of this article demonstrate this conclusion.

### 3.4. Weight Estimation of Features

Feature weighting technique based on certain principle gives a weight to various data features where calculating $\vec{\omega }$ is the key element. The changes in facial expression lead to slight different instant changes in individual facial muscles in facial appearance. According to motion range of facial muscles, the whole face can be divided into three kinds of regions: rigid region (nose), semirigid region (eyes, forehead, and cheek), and on-rigid region (mouth). According to the principles above, we divide face into several areas and find out recognition rate *p*_*i*_  (1 ≤ *i* ≤ *n*) of all the areas where the higher the recognition rates, the greater the influences. Otherwise, the lower the recognition rate, the smaller the influences. Regard weight determination as the base for calculating the value of weight, and the calculation formula is presented as follows:

(16) $$\begin{array}{c} \omega_{i}=\frac{p_{i}}{p_{1}+p_{2}+p_{3}+p_{4}}. \end{array}$$

This approach makes *ω*_1_ + *ω*_2_ + *ω*_3_ + *ω*_4_ = 1.

The area of the highest value of weight has the highest differentiation in the face, although it is also the largest contributor to classification results. Therefore, the higher the value of weight as a correlation measurement index, the stronger the correlation. The four constructing steps of weighted feature SVM are as follows:

(1) Collecting origin facial expression image dataset *O* and extracting feature dataset $S=\left\{d,\vec{f}\right\}$, where $\vec{f}=\left({\vec{f}}^{1},{\vec{f}}^{2},\ldots ,{\vec{f}}^{n}\right)$ is feature vector of facial expression, ${\vec{f}}^{i}\;\;\left(1\leq i\leq n\right)$ is the feature vector of *i*th region of face, and *d* is the corresponding class label of facial expression
(2) Calculating recognition rates *p*_*i*_  (1 ≤ *i* ≤ *n*) and corresponding value of weight *ω*_*i*_ of each area. Constructing feature weight vector $\vec{\omega }$ and linear transformation square matrix *P*, where $P=\mathrm{diag}\left(\vec{\omega }\right)$
(3) Replacing standard kernel function formulas with weighted feature kernel function formulas (12)–(14), and constructing a classifier based on sample dataset *S*
(4) Evaluating the performance of achieved classifier

## 4. Experiment

The experiments on the extended Cohn-Kanade (CK+) dataset show the effectiveness of the proposed method. In our experiments, we use python programs based on LIBSVM software packages, and the platform of data processing is a computer with Windows 7, Intel® Core™ i3-2120 CPU (3.30 GHz), 4.00 GB RAM.

### 4.1. Extended Cohn-Kanade Dataset

 Lucey et al. [36] presented the CK+ dataset containing 593 sequences from 123 subjects. Each of the sequences incorporates images from onset (neutral frame) to peak expression (last frame). But, only 327 of the 593 sequences were found to meet criteria for one of seven discrete emotions. And, 327 peak frames have been selected and labeled which come together to compose origin facial expression image dataset *O*. The detailed number of images of each discrete emotion is shown in Table 1.

### 4.2. Facial Feature Extraction

**Figure 1:**
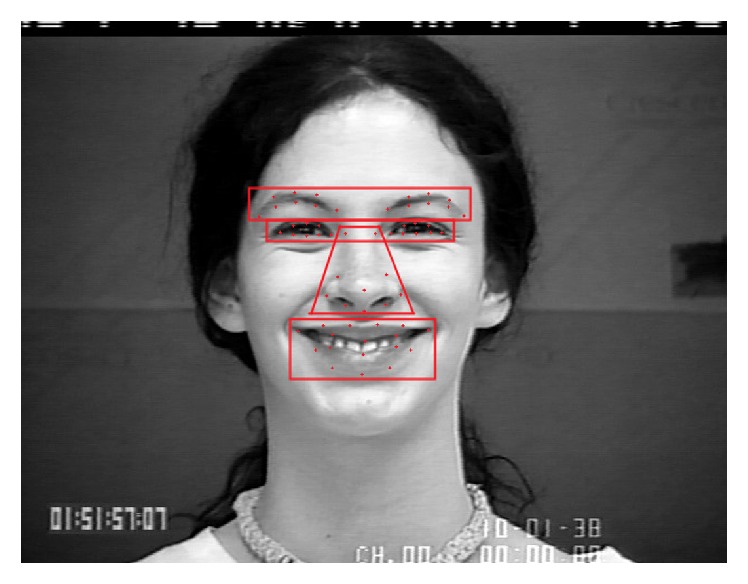
Partition and key points of human face.

In the paper, we use facial key points of each image as feature points on emotion recognition based on facial expression. Each feature point is expressed as a 2-dimensional coordinate as follows: (*x*, *y*). The resolution of each image of dataset *O* is 640 × 490, 640 × 480, or 720 × 480. In order to unify the standard of coordinate system, image preprocessing is used to change the resolution of each image into 640 × 480. Reference [11] proposed the production of emotion, which has brought about facial behavior changes and is strongly linked to not the whole face but some specific areas, such as eyebrows, eyes, mouth, nose, and tissue textures. Besides, a face has different rigidness in different areas. According to the principles above, this paper divides face into 4 areas, which are shown in Figure 1 and corresponding feature vectors are listed as follows.

(1*) Eyebrows Area*. Select 8 key points from each eyebrow; their 2-dimensional coordinates (*x*_1,*k*_, *y*_1,*k*_), *k* = 1,…, 16, work together to form a 32-dimensional feature vector ${\vec{f}}^{1}=\left(x_{1,1},y_{1,1},x_{1,2},y_{1,2},\ldots ,x_{1,16},y_{1,16}\right)$. 

(2*) Eyes*. Select 8 key points from each eye; their 2-dimensional coordinates (*x*_2,*k*_, *y*_2,*k*_), *k* = 1,…, 16, work together to form a 32-dimensional feature vector ${\vec{f}}^{2}=\left(x_{2,1},y_{2,1},x_{2,2},y_{2,2},\ldots ,x_{2,16},y_{2,16}\right)$. 

(3*) Nose*. Select 10 key points from nose; their 2-dimensional coordinates (*x*_3,*k*_, *y*_3,*k*_), *k* = 1,…, 10, work together to form a 20-dimensional feature vector ${\vec{f}}^{3}=\left(x_{3,1},y_{3,1},x_{3,2},y_{3,2},\ldots ,x_{3,10},y_{3,10}\right)$.

(4*) Mouth*. Select 17 key points from mouth; their 2-dimensional coordinates (*x*_4,*k*_, *y*_4,*k*_), *k* = 1,…, 17, work together to form a 34-dimensional feature vector ${\vec{f}}^{4}=\left(x_{4,1},y_{4,1},x_{4,2},y_{4,2},\ldots ,x_{4,17},y_{4,17}\right)$.

Above all, we select 59 key points from the eyebrows, eyes, nose, and mouth. Therefore, 118-dimensional facial feature vector $\vec{f}$ can be got from each frame where $\vec{f}=\left({\vec{f}}^{1},{\vec{f}}^{2},{\vec{f}}^{3},{\vec{f}}^{4}\right)$.

### 4.3. Experiment Contrast with Different Feature

Sample set *S* contains 327 feature vectors of facial images of seven discrete emotions. We use the method of stratification sampling to get training set and test set. First, we treat the sample set *S* in 7 disjoint layers on the basis of certain emotions. Then, we select a fixed number of feature vectors from each layer independently and randomly. The number is determined by the smallest size of 7 facial expression sample sets, which is 70% of the size of contempt sample set in this article. At last, all these selected feature vectors come together to compose training set *T*, while the rest of feature vectors come together to compose test set *V*. The detailed number of feature vectors of each emotion is shown in Table 1.

Select the ${\vec{f}}^{i}$ component of feature vector $\vec{f}$ to compose training set *T*_*i*_  (1 ≤ *i* ≤ 4) and test set *V*_*i*_  (1 ≤ *i* ≤ 4). Thus we experiment four times under different facial area features, respectively. The detailed recognition accuracy of each facial area feature is shown in Table 2. According to the analysis of experimental results in four feature areas, the influence of features of three types of region is different. The nonrigid region has the biggest impact; rigid region has the least while semirigid region has an impact at a fair level.

### 4.4. Experiment Contrast with Different Kernel Function

We use the previous experiment results and (10) and (16) to obtain the weight *ω*_*i*_ of each area and corresponding linear transformation square matrix *P* as follows:

(17) ω→ω1,ω2,ω3,ω4=0.24,0.25,0.15,0.36,PP10000P20000P30000P4=ω1E32×320000ω2E32×320000ω3E20×200000ω4E34×34.

Standard Gaussian kernel function $k\left({\vec{f}}_{i},{\vec{f}}_{j}\right)$ and weighted feature Gaussian kernel function $k_{P}\left({\vec{f}}_{i},{\vec{f}}_{j}\right)$ can be got by (7) and (13) for the 118-dimensional facial feature vector $\vec{f}$

(18) kf→i,f→jexp⁡−γf→i−f→j2,kPf→i,f→jexp⁡−γf→iTP−f→jTP2=exp⁡−γf→i−f→jTPPTf→i−f→j.

Thus we experiment twice under training set *T* and test set *V* with different kernel function, respectively. The number of correctly recognized facial expressions under two kernel functions is shown in Table 3.

Finally, we compare our results with the experiments of two kernel functions, which are all image-based framework and tested on the CK+ dataset. The average precision of WF-SVM which uses weighted feature Gaussian kernel function is 93%, which is higher than SVM that uses standard Gaussian kernel function whose average precision is 83%, as is shown in Table 3. And the recognition rate is better than the previous method for the seven emotions. These confirm the effectiveness of our method. After investigating the reason, we find it can be explained from robustness of machine learning algorithm. This method reduces the influence of weak correlation feature by weighted feature, thus improving the robustness of algorithm.

## 5. Conclusion and Future Work

In this paper, we propose an approach of emotion recognition based on facial expression. In our approach, we propose a feature weighting technique since the effect of each feature on recognition result is different. Different from previous works by calculating weight of each feature directly, the facial expression images are divided into some uniform subregions and weight of subregion features is calculated based on their respective subrecognition rate. The experimental results suggest that the approach based on weighted feature Gaussian kernel function has good performance on the correct rate in emotion recognition. But our approach shows a pretty good performance for the dataset with limited head motion. Emotion recognition based on facial expression is still full of challenges in the future.

## Figures and Tables

**Table 1:** The detailed number of images of each discrete emotion in dataset *O*.

	Sample set	Training set	Test set
Anger	45	13	32
Contempt	18	13	5
Disgust	59	13	46
Fear	25	13	12
Happiness	69	13	56
Sadness	28	13	15
Surprise	83	13	70

**Table 2:** The recognition accuracy of each facial area feature.

Subregion	Eyebrows	Eyes	Nose	Mouth
Recognition rate	40.55%	41.94%	25.45%	60.37%

**Table 3:** The number and average precision of correctly recognized facial expressions under two kernel functions.

Emotion	Test set	SVM	WF-SVM
Anger	32	24	28
Contempt	5	2	4
Disgust	46	40	42
Fear	12	9	11
Happiness	56	50	54
Sadness	15	10	12
Surprise	70	62	69
Average precision	—	83%	93%
